# Interactions between Sirt1 and MAPKs regulate astrocyte activation induced by brain injury in vitro and in vivo

**DOI:** 10.1186/s12974-017-0841-6

**Published:** 2017-03-29

**Authors:** Dan Li, Nan Liu, Hai-Hua Zhao, Xu Zhang, Hitoshi Kawano, Lu Liu, Liang Zhao, Hong-Peng Li

**Affiliations:** 1 Department of Human Anatomy, College of Basic Medical Sciences, China Medical University, Shenyang, China; 2 Department of Health and Dietetics, Faculty of Health and Medical Science, Teikyo Heisei University, Tokyo, 170-8445 Japan

**Keywords:** Brain injury, Sirt1, ERK pathway, JNK pathway, p38MAPK pathway, Astrocyte activation

## Abstract

**Background:**

Astrocyte activation is a hallmark of traumatic brain injury resulting in neurological dysfunction or death for an overproduction of inflammatory cytokines and glial scar formation. Both the silent mating type information (Sirt1) expression and mitogen-activated protein kinase (MAPK) signal pathway activation represent a promising therapeutic target for several models of neurodegenerative diseases. We investigated the potential effects of Sirt1 upregulation and MAPK pathway pharmacological inhibition on astrocyte activation in vitro and in vivo. Moreover, we attempted to confirm the underlying interactions between Sirt1 and MAPK pathways in astrocyte activation after brain injury.

**Methods:**

The present study employs an interleukin-1β (IL-1β) stimulated primary cortical astrocyte model in vitro and a nigrostriatal pathway injury model in vivo to mimic the astrocyte activation induced by traumatic brain injury. The activation of GFAP, Sirt1, and MAPK pathways were detected by Western blot; astrocyte morphological hypertrophy was assessed using immunofluorescence staining; in order to explore the neuroprotective effect of regulation Sirt1 expression and MAPK pathway activation, the motor and neurological function tests were assessed after injury.

**Results:**

GFAP level and morphological hypertrophy of astrocytes are elevated after injury in vitro or in vivo. Furthermore, the expressions of phosphorylated extracellular regulated protein kinases (p-ERK), phosphorylated c-Jun N-terminal kinase (p-JNK), and phosphorylated p38 activation (p-p38) are upregulated, but the Sirt1 expression is downregulated. Overexpression of Sirt1 significantly increases the p-ERK expression and reduces the p-JNK and p-p38 expressions. Inhibition of ERK, JNK, or p38 activation respectively with their inhibitors significantly elevated the Sirt1 expression and attenuated the astrocyte activation. Both the overproduction of Sirt1 and inhibition of ERK, JNK, or p38 activation can alleviate the astrocyte activation, thereby improving the neurobehavioral function according to the modified neurological severity scores (mNSS) and balance latency test.

**Conclusions:**

Thus, Sirt1 plays a protective role against astrocyte activation, which may be associated with the regulation of the MAPK pathway activation induced by brain injury in vitro and in vivo.

## Background

Traumatic brain injury can be a serious insult caused by a wide variety of stimuli, enclosing a large range of severities [1]. These severities not only induce direct and immediate neuronal injury but also have the potential for long-term and gradually evolving sequelae, such as overactivation of glial cells and secretion of circulating leucocytes around the lesion site, contributing to the failure of functional recovery. Astrocytes tile the brain and localize to all cellular environments, which are the key players in response to injury [2, 3]. In an environment encompassing a wide range of neuroinflammatory factors such as IL-1β induced by central nervous system (CNS) injury, the astrocytes are transformed as “activated” [4]. The activated astrocytes produce multiple molecular and morphological features that have been considered as hallmarks of astrocyte activation in response to CNS injury by histopathologists and researchers [5–9]. The most prominent of these hallmarks are hypertrophy of astrocyte cellular processes and upregulation of the glial fibrillary acidic protein (GFAP), which is the main constituent of the intermediate filament system of adult astrocytes [10]. Several studies have shown that GFAP upregulation accompanied hypertrophy and migration of astrocytes after a traumatic brain injury [11, 12]. Our previous study also confirmed that the downregulation of GFAP expression could attenuate the astrocyte activation after brain injury [13], suggesting that its upregulation is crucial for astrocyte activation.

Sirtuins are mammalian homologs of the *Saccharomyces cerevisiae* silent mating type information 2 (Sirt2), which are members of the class III histone/lysine deacetylase family. The Sirts use NAD^+^ as an obligatory co-substrate to remove an acety1 group from the epsilon amine of lysine [14, 15]. *Sirt1* is the first homologous gene of this family identified in mammals. Some recent studies have found that the pharmacological activation or upregulation of Sirt1 expression showed neuroprotective effects in several models of neurodegenerative diseases [15–18]. Notably, Sirt1 is widely expressed in the entire adult brain [19] and involved in the maintenance of brain integrity regulating activities such as oxidative stress and neuronal degeneration [20]. However, the underlying roles of Sirt1 in astrocyte activation after brain injury are yet ill-understood.

Our previous studies found that mitogen-activated protein kinase (MAPK) cascades were involved in the glial activation [13] and resveratrol protects against striatum neuronal apoptosis induced by a nigrostriatal pathway injury in mice via MAPK pathway [21]. Moreover, some studies showed that Sirt1 participates in learning and memory through MAPKs. Sirt1 inhibition reduced the Ras/ERK1/2 pathway associating with resistance to oxidative damage, suggesting that a correlation between Sirt1 and MAPK pathways to protect against the central nervous system (CNS) injury through yet unknown mechanisms [22–25].

Thus, we hypothesize that both the Sirt1 and MAPK pathways, such as ERK, JNK, and p38, were involved in regulating the astrocyte activation, and some networks through yet unknown mechanisms promote the recovery of neural function via attenuation of the astrocyte activation. To test this hypothesis, we applied a nigrostriatal pathway injury in the mouse brain to mimic the traumatic brain injury in vivo and IL-1β stimulation model to induce astrocyte activation in vitro. Based on these in vitro and in vivo models, we analyzed the expression of Sirt1 and p-ERK, p-JNK, and p-p38 after stimulation or injury and monitored GFAP expression and astrocytes hypertrophy, as well as the interactions between Sirt1 and MAPK (ERK, JNK, p38MAPK) pathways after manipulation of Sirt1 and p-ERK, p-JNK, and p-p38 levels pharmacologically and genetically.

## Methods

### Reagents

Human recombinant IL-1β (1 × 10^9^ IU/mg protein) was purchased from R & D Systems (Jiangsu, China). The cytokine was prepared as the previous study [4]. Medium, fetal bovine serum and penicillin-streptomycin solution were from Gibco-BRL/Thermo Fisher (Co., Ltd., USA); resveratrol (3,4,5′-trihydroxy-trans-stilbene) and dimethyl sulfoxide (DMSO) were provided by Sigma-Aldrich Inc. (St. Louis, MO, USA). U0126, SP600125, and SB203580 from Selleck. cn (Shanghai, China) were dissolved in 0.1% DMSO, which was used as a solvent control. Primary antibodies including Sirt1 and mitogen-activated protein kinases (MAPKs) proteins such as p-ERK, JNK, and p38 MAPK were obtained from CST (Shanghai, China), t-ERK/JNK/p38 MAPK from Proteintech (Wuhan, China), GFAP from Millipore (MA, USA), and GAPDH from Abcam (Shanghai, China). The secondary antibody for Western blot was HRP-conjugated anti-rabbit lgG from Zhongshan Company (Shanghai, China). Please contact the author for more detailed data requests.

### Primary culture of mouse cortical astrocytes

Primary astrocytes were prepared from 1-day-old neonatal Kunming mice as described previously with some modifications [26]. Briefly, brain cortical tissues were dissociated in DMEM supplemented with 10% fetal bovine serum, 100 U/ml penicillin, and 100 μg/ml streptomycin. Cells were incubated at 37 °C for 24 h in 95% air and 5% CO_2_. The medium was changed after 5 days and every 3 days thereafter. The cell cultures were used 9 days after plating. For pure astrocyte culture, astrocytes were isolated from culture flasks of confluent glial cultures by shaking at 250 rpm for 24 h. Astrocytes in the supernatant were collected by centrifugation at 1000 rpm for 5 min. Purified astrocytes were seeded into 24-well plates at 1 × 10^5^ cells/ml. The purity of astrocytes was greater than 95% as determined by immunostaining using the marker of astrocyte glial fibrillary acidic protein (GFAP). The astrocytes were stimulated with 1 ng/ml IL-1β for the tested time course [4]. Cultures were pretreated with 10 μM U0126 (blocks the ERK pathway), 10 μM SP600125 (blocks the JNK pathway), 10 μM SB203580 (blocks the p38MAPK pathway), or DMSO (as a vehicle) for 30 min in serum-free media before stimulated with IL-1β.

### Animal preparation

All experimental procedures were conducted in conformity with institutional guidelines for the care and use of laboratory animals, and protocols were approved by the Institutional Animal Care and Use Committee (CMU IACUC) in China Medical University (Shenyang, China). Male KM mice (CL) 8-week-old (25–30 g) were purchased from the Animal Department, China Medical University (Shenyang, China), and kept under a constant environment (12/12-h light/dark cycle). They were allowed free access to water and food. All the animals were randomly assigned into the following group: (i) sham injury, (ii) brain injury+DMSO, and (iii) brain injury+resveratrol or MAPK (ERK/JNK/p38MAPK) inhibitors. The resveratrol/inhibitors or DMSO was injected intracerebroventricularly 30 min prior to brain injury, respectively, including 3 μg/μl resveratrol [27], 400 μM U0126 (ERK inhibitor), 100 μM SP600125 (JNK inhibitor), and SB203580 (p38 kinase inhibitor) following previous studies [25, 28–30].

### Behavioral testing

All behavioral tests were performed blindly with respect to drug administration. The test was conducted prior to and at 1 and 21 days after injury. Animals were pretrained for 3 days for all the tests. Behavioral studies were repeated two times with three different trials to validate the behavioral data.

The modified neurological severity score (mNSS) is a composite test, including motor, sensory, and reflex tests [31]. A scale of the 0–18 score was graded on the neurological function wherein the normal score was 0 and a maximal deficit score was 18. One point was given for the inability to perform each test or for the absence of a reflex.

Beam walking test—the beam walking test assessed fine motor coordination and function by measuring the ability of the animals to traverse an elevated narrow beam as described previously [32]. The time for the mouse to cross the beam (not to exceed 60 s) was recorded. The measurements were recorded 1 day before the brain injury (as the baseline) and at each tested time point after brain injury for all the tests.

For all the tests, three measurements per trial were recorded 1 day before brain injury (baseline) and at each tested time point after brain injury.

### The model of nigrostriatal pathway injury

The nigrostriatal pathway injury in mouse was used as the traumatic brain injury model. Briefly, the mice were fixed in a stereotaxic apparatus (TME Technology Co., Ltd., Chengdu, China) with the incisor bar set 3 mm below the intraaural line under 10% chloral hydrate (30 mg/kg body weight) anesthesia. The mice were sheared and sterilized at first. The middle skin was incised on the preshaved scalp, periosteum was cleared from the cranium, and a small oval hole was made with a dental drill wherein a 2.0-mm razor blade knife was inserted [33, 34]. A rectangular gap was drilled at 1.5 mm right back of the bregma, exposing the brain. A 2.0-mm width cutter was fixed in a linear type space frame, and the tip of the knife was inserted into the right side of the brain at 0.5 mm lateral to the midline, 1.5 mm posterior to the bregma, and at a depth of 6.0 mm from the surface of the brain. Then, the cutter was slowly pulled out with hemostatic suture. The skull was only exposed in the sham group. For all mice, constant temperature operating table was used in the whole process of brain injury and the room temperature was controlled approximately at 25 °C in an air-conditioned room after the brain injury.

### Adenovirus construction

To evaluate the potential effect of Sirt1 on activation of astrocytes in vitro, the astrocytes were transfected with control or Sirt1 for 24 h and then stimulated with 1 ng/ml IL-1β. The Sirt1-recombined adenoviral expression vector and the control vector were constructed (Invitrogen, Carlsbad, CA, USA) and performed at a multiplicity of infection (MOI) of 20 [35, 36]. The lacZ vector (Invitrogen) was added to control groups.

Cells (1 × 10^6^) grown to 60–80% confluence in 24 wells were transfected with adenoviral vectors using a Lipofectamine 2000 kit (Invitrogen, cat. 11668–019) according to the procedure provided by the manufacturer. The cells were observed under a fluorescence microscope and harvested 24 h after transfection.

### Western blot

Fresh brain tissues from injured part, which the area up and down 1 mm from the center of the lesion site including the nigrostriatal pathway, were taken and placed in cool PBS. The cerebral localization states briefly a rectangular area (2 × 2 mm) is 0.5 mm the posterior, 2.0 mm lateral from the bregma, and 5.5–6.0 mm deep from the cortex [13]. Western blot analysis was performed as previously described [37]. Briefly, equal amounts of protein (35 mg protein in 20 ml for tissue samples) were separated by sodium dodecyl sulfate-polyacrylamide gel, transferred to PVDF membranes (Millipore, US), and blocked using 5% bovine serum albumin (BSA). The primary antibodies were rabbit polyclonal anti-Sirt1 (1:1000), p-ERK/JNK/p38MAPK (1:1000), t-ERK/JNK/p38MAPK (1:2000), and mouse monoclonal anti-GAPDH (1:5000). All the antibodies were diluted by 5% BSA. In the following day, a goat anti-rabbit or goat anti-mouse secondary antibody (1:5000) labeled by horseradish peroxidase was added and incubated for 90 min at 37 °C. Bio-Rad was used for ECL luminescence.

### Immunohistochemistry

Immunohistochemical analyses were performed according to our previous study [13]. Briefly, primary antibodies of chick anti-GFAP were diluted to 1:500 in 20 mM phosphate-buffered salines (PBS) and incubated overnight. In the following day, the sections or the cells were washed by PBS and cy3 (Jackson ImmunoResearch Labs, Donkey anti-Chick, 1:100) was added and incubated for 1 h at 37 °C. After washing by PBS, the fluorescence quenching agent was used to cover the slices. Fluorescent microscope (HMS Nikon Imaging Center, ECLIPSE 80i, Japan) was used to observe and a CCD spot camera was employed to collect photos. The photos were saved as TIF and processed by Adobe Photoshop 7.0 (Adobe Photoshop CS2).

### Quantification and statistics

In the quantitative statistics of Western blot, the detection of gray value in p-ERK/JNK/p-38 bands used computer-assisted imaging analysis system (Bio-Rad) by drawing a rectangle with the same size to detect gray value of p-ERK/JNK/p-38 proteins. Because the total ERK/JNK/p-38 level did not change after the nerve injury, the gray level ratio of p-ERK and t-ERK, p-JNK and t-JNK, p-p38 MAPK and p38 MAPK, and the ratios of Sirt1 and GFAP to GAPDH were used as the relative expression level of the target protein. All the experiments were repeated for at least three times. Data are presented as the mean and standard error of the mean (mean ± SEM). The comparison of different groups was analyzed by one-way ANOVA followed by the post hoc Bonferroni evaluation using GraphPad Prism5. Differences were termed statistically significant at *P* < 0.05.

## Results

### IL-1β-stimulated model and stab brain injury model could induce astrocyte activation

**Fig. 1:**
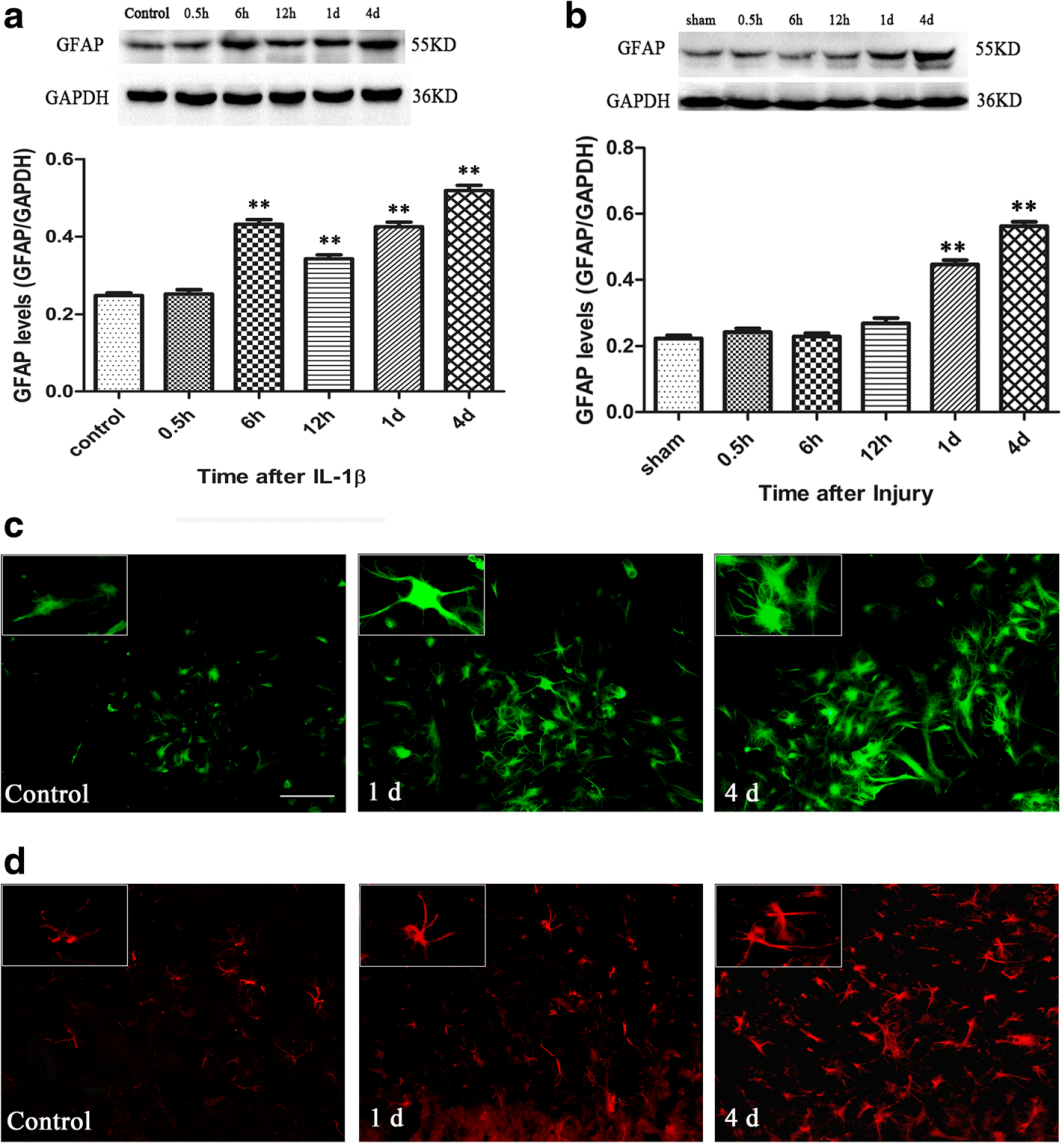
Both the IL-1β stimulation and brain injury could induce the astrocyte activation. **a** Western blot showed that the GFAP expression after IL-1β stimulation, *P < 0.05 and **P < 0.01 vs. the control group, the control group: control without IL-1β stimulation. **b** The GFAP expression was showed after brain injury, *P < 0.05 and **P < 0.01 vs. the sham group. We also detected changes in astrocyte morphology by immunofluorescence staining in vitro; the GFAP-positive astrocytes were *green* (**c**) and in vivo and the GFAP-positive astrocytes were *red* (**d**) at 1 and 4 days, respectively. Scale bar = 50 μm

As shown in Fig. 1a, compared to the control astrocytes, the GFAP expression was significantly increased within 0.5 h of IL-1β stimulation, indicating IL-1β-induced astrocyte activation. Compared with the control, the GFAP-positive astrocytes begin to proliferate with phenotypic changes and cell hypertrophy and the characteristics of the latter depend on the increased expression of GFAP in 1- and 4-day groups after IL-1β stimulation (Fig. 1c). In vivo, the protein level of GFAP was significantly increased at 1 and 4 days after brain injury. Compared with the sham group, the astrocytes became thicker and cell hypertrophy was noticeable around the lesion site at 1 and 4 days after brain injury (Fig. 1d). These results indicated that brain injury could induce astrocyte activation around the lesion site.

### IL-1β-stimulated model and stab brain injury model could reduce the expression of Sirt1 and induce the activation of MAPK pathways

**Fig. 2:**
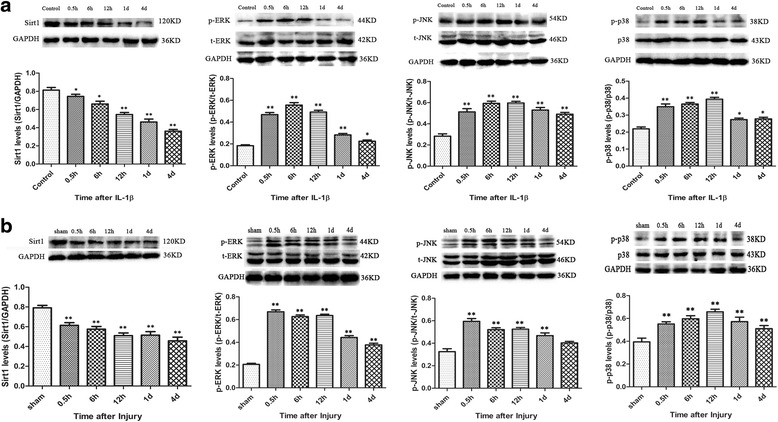
The expression of Sirt1 and p-ERK/p-JNK/p-p38 after IL-1β stimulation and brain injury. The expression of Sirt1 is reduced and p-ERK, p-JNK, and p-p38 are elevated significantly for the time indicated in vitro (**a**) and in vivo (**b**). *n* = 6/group, *P < 0.05 and **P < 0.01 vs. the control or sham group

The results showed that the treatment with IL-1β significantly reduced the expression of Sirt1 protein from 0.5 h to 4 days. Concurrently, the ERK, JNK, and p38MAPK pathways were activated from 0.5 h and continued until 4 days. In addition, we also found that the p-ERK, p-JNK, and p-p38 proteins were at a high level within 1 day (Fig. 2a).

Compared with the sham group, the expression of Sirt1 in the injured groups was significantly decreased from 0.5 h to 4 days after brain injury (Fig. 2; *P* < 0.01 for all the evaluated time points). ERK, JNK, and p38MAPK pathways were activated significantly from 0.5 h and lasted for 4 days after brain injury compared with sham groups (*P* < 0.01 for all the evaluated time points). These results revealed that Sirt1 and MAPK pathways including ERK, JNK, and p38MAPK were also involved in the mechanism of astrocyte activation induced by brain injury, which was consistent with the in vitro results.

### Sirt1 overexpression by transfected Sirt1 or pharmacological agonist resveratrol decreased the astrocyte activation in vitro and in vivo

**Fig. 3:**
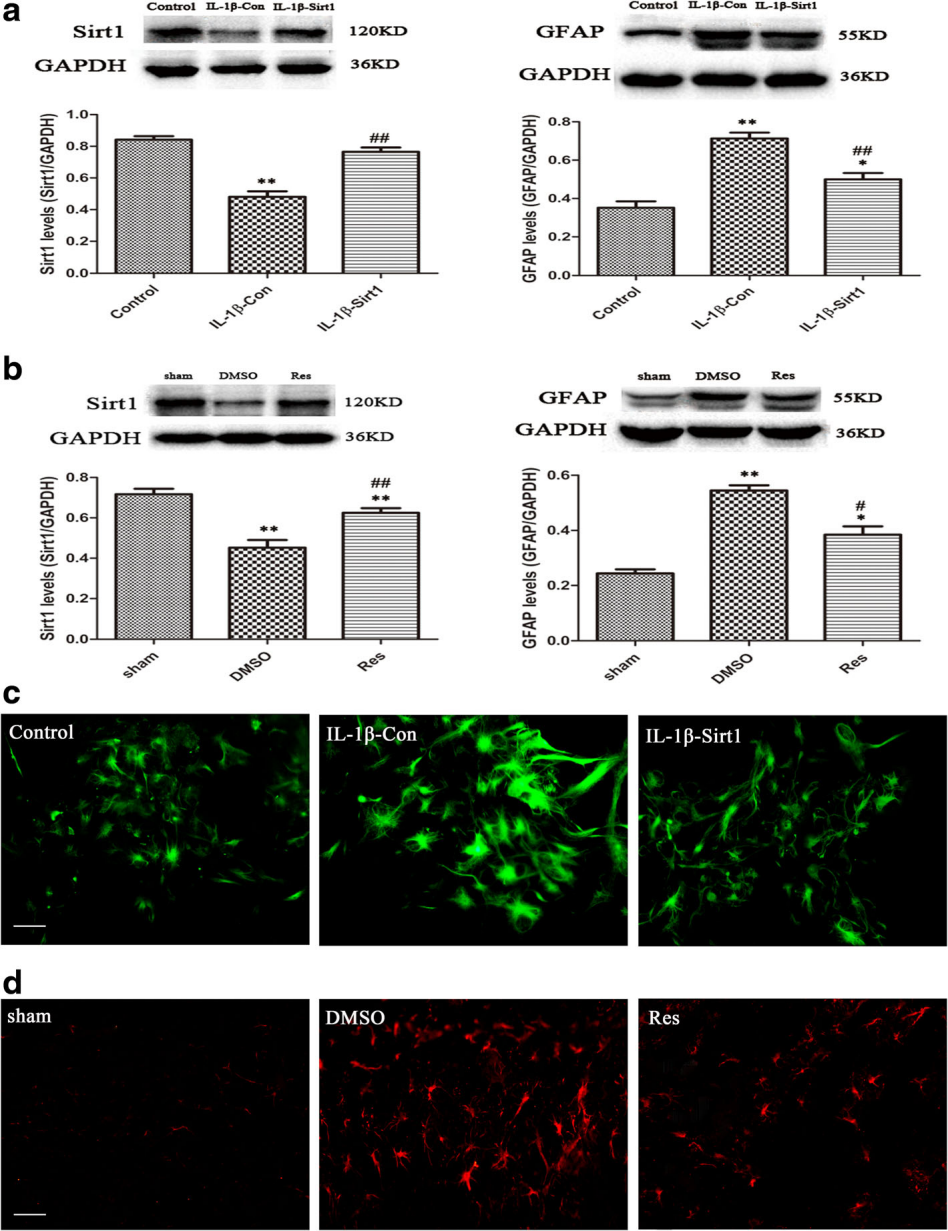
Overexpression of Sirt1 attenuates astrocyte activation in vitro and in vivo. Overexpression of Sirt1 significantly reduced GFAP expression compared with transfection control in vitro (**a**), *n* = 6/group, **P < 0.01 vs. the control group; ^##^ *P* < 0.01 vs. IL-1β-con; and in vivo (**b**), *P < 0.05 and **P < 0.01 vs. the sham group; ^#^ *P* < 0.05 and ^##^ *P* < 0.01 vs. the DMSO group. Immunofluorescence staining revealed that overexpression of Sirt1 improved the signs of astrocyte hypertrophy by immunodetection of GFAP in vitro (**c**, *green*) and in vivo (**d**, *red*)

The results revealed that the primary astrocytes transfected with Sirt1 appeared to elevate the Sirt1 expression and decrease the GFAP expression significantly as compared to those transfected with control vector after 24 h (Fig. 3a). As shown in Fig. 3c. As compared to the transfection with control, the Sirt1-transfected astrocytes showed fewer signs of cell hypertrophy and were similar to the control group.

As shown in Fig. 3b, as compared to pretreating with DMSO, pretreatment with resveratrol significantly increased the expression of Sirt1 at 1 day and reduced the expression of GFAP at 4 day after brain injury. Immunofluorescence staining revealed that resveratrol could remarkably improve the signs of astrocyte hypertrophy around the lesion site (Fig. 3d). The results suggested that Sirt1 might function as a protective factor for astrocyte overactivation after brain injury in vivo.

### Sirt1 overexpression upregulated ERK activation and downregulated JNK and p38MAPK activation induced by IL-1β-stimulation in vitro and brain injury in vivo

**Fig. 4:**
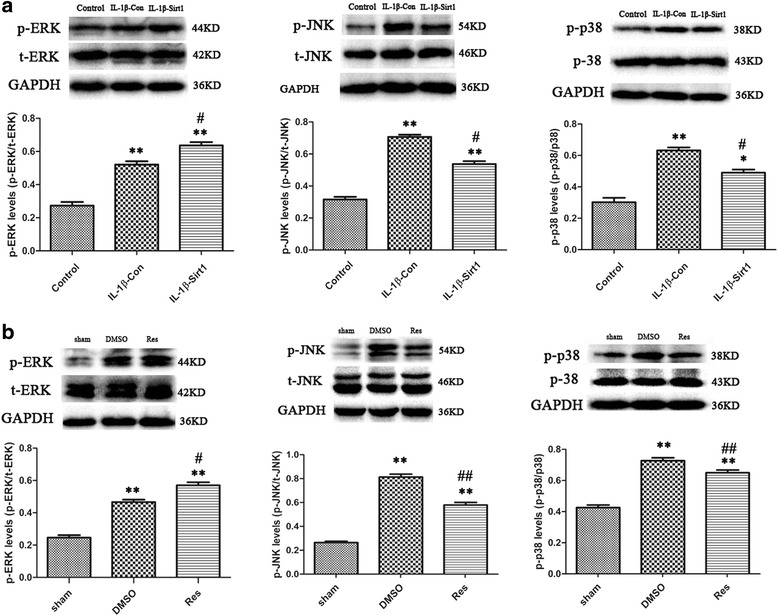
The effects of Sirt1 overexpression on regulation of p-ERK/p-JNK/p-p38 in vitro and in vivo. **a** Overexpression of Sirt1 upregulates p-ERK and downregulates p-JNK and p-p38 compared to transfection control in vitro. *n* = 6/group, **P < 0.01 vs. the control group; ^#^ *P* < 0.05 vs. IL-1β-con. **b** Elevated Sirt1 expression increased ERK activation and decreased JNK and p-38 activation in vivo. *n* = 6/group, **P < 0.01 vs. the sham group; ^#^ *P* < 0.05 and ^##^ *P* < 0.01 vs. the DMSO group

Compared with the transfection control, the results showed that transfected Sirt1 markedly upregulated the p-ERK expression and downregulated the p-JNK and p-p38MAPK expressions at 1 day after IL-1β stimulation in astrocytes (all *P* < 0.05; Fig. 4a). This suggested potential interactions between Sirt1 and MAPKs regulating the astrocyte activation induced by IL-1β stimulation.

The expressions of p-ERK, p-JNK, and p-p38 at 1 day after brain injury were shown in Fig. 4b. When the resveratrol-treated group significantly elevated the Sirt1 expression (*P* < 0.01; Fig. 3b), p-ERK expression was markedly increased and p-JNK/p-p38 MAPK expressions were reduced (*P* < 0.05 for p-ERK, *P* < 0.01 for p-JNK and p-p38MAPK; Fig. 4b), as compared to the treatment with DMSO in mice after brain injury. The results indicated that Sirt1 mediated the expression of ERK, JNK, and p-38MAPK pathways after mechanical brain injury in vivo.

### The effects of inhibitors of MAPK pathways on ERK/JNK/p38 activation

**Fig. 5:**
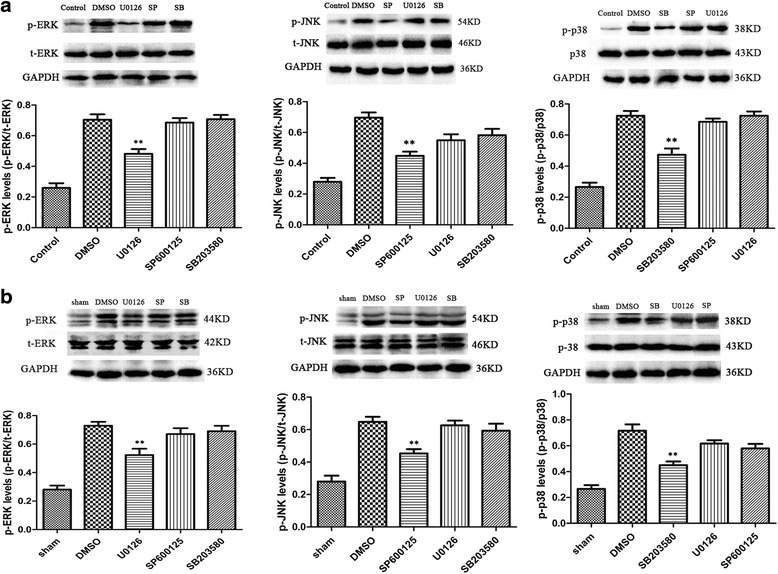
The effects of inhibitors U0126, SP600125, and SB203580 on ERK, JNK, and p38 activation. **a** U0126, SP600125, and SB203580 (10 μM) significantly inhibited ERK, JNK, and p38MAPK activation respectively in vitro. *n* = 6/group, **P < 0.01 vs. the DMSO group. **b** U0126 (200 μM), SP600125, and SB203580 (100 μM) significantly inhibited ERK, JNK, and p38MAPK activation respectively around the lesion site in vivo. *n* = 6/group, **P < 0.01 vs. the DMSO group

As shown in Fig. 5a, when the ERK activation was inhibited by U0126, the JNK and p38 MAPK activation were not affected. Similarly, SP600125 or SB203580 could inhibit the JNK activation or p38MAPK activation, respectively, but did not affect the other two pathways.

The results showed that U0126 significantly inhibited the ERK activation but exerted no effect on the JNK or p38MAPK activation in comparison to the DMSO injection (*P* < 0.05 for ERK; *P* > 0.05 for JNK and p38MAPK; Fig. 5b). Similarly, P600125 significantly inhibited the JNK activation without affecting the ERK or p38MAPK activation as compared to the DMSO injection (*P* < 0.05 for JNK; *P* > 0.05 for ERK and p38MAPK; Fig. 5b). SB203580 significantly inhibited the p38MAPK activation but showed no effects on ERK or JNK activation as compared to DMSO injection (*P* < 0.05 for p38MAPK; *P* > 0.05 for ERK and JNK; Fig. 5b).

### Inhibiting ERK, JNK, or p38MAPK pathway activation elevated Sirt1 expression and attenuated astrocyte activation in vitro and in vivo

**Fig. 6:**
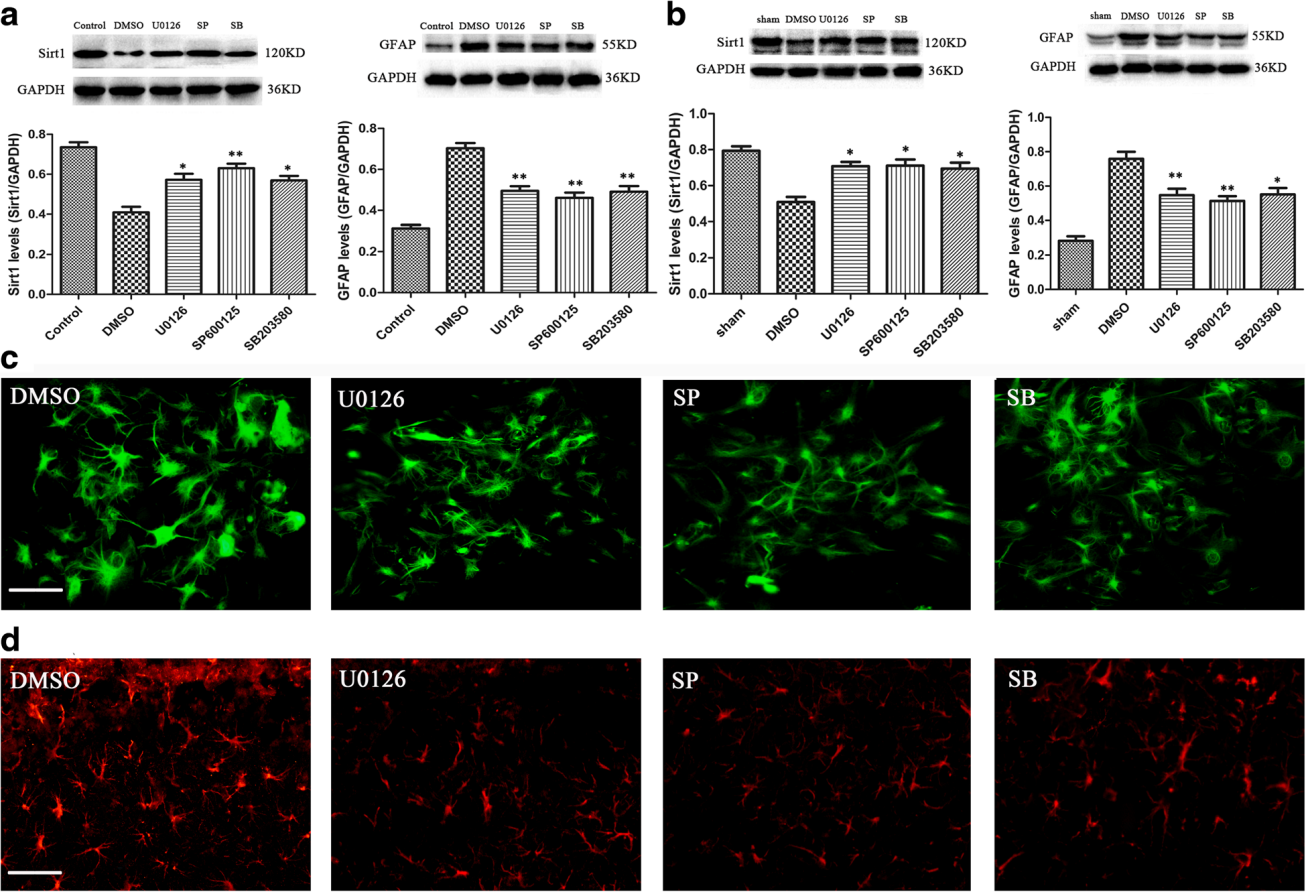
Inhibiting MAPK pathway activation increases Sirt1 expression and alleviates astrocyte activation. **a** Levels of Sirt1 and GFAP protein in primary astrocytes after IL-1β stimulation in vitro. **b** Inhibiting MAPK pathway activation significantly increases Sirt1 expression but alleviates GFAP expression in vivo. *n* = 6/group, *P < 0.05 and **P < 0.01 vs. the DMSO group. Immunofluorescent image of GFAP in vitro and in vivo was showed in (**c**) and (**d**). Scale bar = 50 μm

As shown in Fig. 6a, when the ERK, JNK, or p38MAPK pathway was inhibited at 1 day after IL-1β stimulation, the protein level of Sirt1 was significantly elevated in the primary astrocytes treated with the inhibitor as compared with the treatment with DMSO (*P* < 0.05 for U0126 and SB203580; *P* < 0.01 for SP600125). Moreover, Western blot demonstrated that the inhibition of ERK, JNK, or p38MAPK pathway significantly suppressed the GFAP protein level at 4 day after IL-1β stimulation in primary astrocytes (*P* < 0.01 for all; Fig. 6a). However, the DMSO control did not show any effect on GFAP protein level as compared to the primary astrocytes stimulated with IL-1β (*P* > 0.05, data not shown).

To further substantiate the in vitro finding, a stab wound brain injury was employed. When the ERK, JNK, or p38MAPK pathway was inhibited, the expression of Sirt1 was significantly elevated (*P* < 0.05 for all the inhibitors; Fig. 6b) and the GFAP level was attenuated as compared to DMSO (*P* < 0.05 for SB203580; *P* < 0.01 for U0126 and SP600125; Fig. 6b). We also detected a significant suppression of astrocyte hypertrophy and GFAP staining by immunofluorescence in vitro and in vivo (Fig. 6c, d).

### Elevating Sirt1 expression and inhibiting ERK, JNK, or p38MAPK pathway activation improved the neurobehavioral function after brain injury

**Fig. 7:**
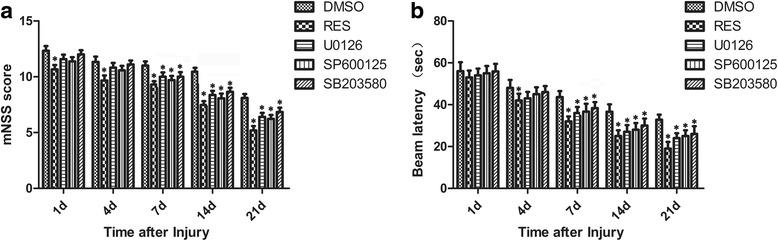
The neuroprotection in mice pretreated with resveratrol to elevate Sirt1 expression or inhibitors to inhibit MAPK pathway activation. **a** The mNSS significantly reduced in mice with resveratrol and inhibitor groups. **b** Beam latency showed that resveratrol and inhibitor groups significantly decreased the time of mice to cross the beam compared to the DMSO group after brain injury. Values are showed as mean ± SEM. *n* = 6/group, *P < 0.05 vs. the DMSO group

As shown in Fig. 7a, the mean mNSS was significantly reduced in the resveratrol group (from 1 to 21 days all *P* < 0.05), U0126 group (from 4 to 21 days, *P* < 0.05), SP600125 group (from 7 to 21 days, *P* < 0.05), and SB203580 group (from 7 to 21 days, *P* < 0.05) as compared to the DMSO group. Moreover, the beam walk performances also showed significantly reduced latency to cross the beam from 4 to 21 days after injury (from 4 to 21 days, *P* < 0.05 for the resveratrol group; from 7 to 21 days, *P* < 0.05 for the U0126 group; from 7 to 21 days, *P* < 0.05 for the SP600125 group; from 7 to 21 days, *P* < 0.05 for the SB203580 group; Fig. 7b) as compared to the DMSO group.

## Discussion

Astrocyte activation is presumed to depress neuronal regeneration after CNS injury due to the glial scar, a formation of a physical barrier, and overproduction of multiple proinflammatory cytokines, including IL-1β, IL-6, and TNFα, which further aggravate the glial activation and injure the remaining neurons through positive feedback [4, 31, 38, 39]. The recombinant IL-1β used in the present study was shown to be biologically active as previously demonstrated by its ability to induce astrocyte activation in an in vitro astrocyte activation model [4, 40–42]. Therefore, we speculate that our IL-1β stimulation model is suitable and credible for the detection of the astrocyte activation in vitro. Upregulation of GFAP and hypertrophy of astrocyte cellular processes play a major and prominent role in astrocyte activation and the formation of glial scar [6, 12]. In the present study, the IL-1β stimulation triggered an elevated level of GFAP and induced the astrocyte hypertrophy; this phenomenon was confirmed by the lesion site in a traumatic brain injury. The rational design of therapeutic interventions for CNS injury could be contingent critically upon a comprehensive understanding of the process of astrocyte activation, thereby necessitating an exploration of the mechanism of GFAP upregulation and astrocyte cellular hypertrophy after brain injury.

In recent years, Sirt1 has emerged as a strategy for the treatment of multiple neurodegenerative disorders [25, 43–46]. Although highly expressed in the brain, Sirt1 is also known to localize in the neurons and glial cells among various cell types [47–50]. In addition, investigators found that the expression of Sirt1 varies in different tissues or cell types’ response to injury; some supported that the injury could induce Sirt1 activation and upregulate its expression [17, 25, 51], and others showed that Sirt1 expression was downregulated after the injury [52]. In the present study, we reported that the expression of Sirt1 was downregulated after brain injury in vivo; a similar result was obtained in primary astrocytes after stimulation with IL-1β in vitro. Moreover, the administration of resveratrol, a pharmacological Sirt1 agonist, attenuated astrocyte activation by decreasing the GFAP expression and improving the cell morphology in vivo. The overexpression of Sirt1 in primary astrocytes transfected with lentiviral vector Sirt1 in vitro also generated a similar result. The evidence suggests that Sirt1 expression was involved in the setting of astrocyte activation after injury in vivo or stimulation in vitro, which strongly supports the mechanism of the neuroprotective effect of Sirt1.

Previous study showed that overactivated astrocyte requires energy from mitochondrial biogenesis and the overenhanced mitochondrial respiration in astrocytes limits the substrate supply from astrocytes to neurons [53]. Recently, it was suggested that neurons can release and transfer damaged mitochondria to astrocytes for disposal and recycling [54]. Mitochondrial function may be essential for these neuroglial protective mechanisms because damage of astrocytic mitochondria makes neurons vulnerable to cell death [55]. It may be surmised that increased mitochondrial function could support the functionality of astrocytes. As one of the major targeting organelles of Sirt1 (through PGC1α), mitochondrial function would be a highly possible candidate connection in neuroprotection of Sirt1. Resveratrol, one of the Sirt1 agonists, improves mitochondrial function and protects against metabolic disease by activating Sirt1 and PGC-1α [56]. In this study, we found that increased Sirt1 expression could attenuate the astrocyte activation and improve the neurobehavioral function after brain injury, the mechanism of which may be related to effects of Sirt1 on mitochondrial function in astrocytes. Further experiments are needed to verify this hypothesis.

After brain injury, MAPK family, including ERK, JNK, and p38MAPK, are highly expressed and inhibiting either of the cascades could alter the outcome of the brain injury [13, 57–61]. Though the ERK, JNK, and p38MAPK activation are proven to participate in a variety of injuries in vitro and in vivo, the roles of these three cascade activation in astrocyte stimulation in vivo and in vitro are yet controversial, which leads to the difficulties in exploring their effects on the mechanism of brain injury. Initially, our previous study has shown disparate roles of ERK stimulation in glial activation after brain injury in vivo [13]; however, some studies demonstrated that ERK activation also contributed to neuroprotection [62, 63]. Gao et al. (2013) identified that the inhibition of JNK, but not ERK and p38, suppressed GFAP upregulation in an astrocyte-scratched injury model in vitro, implying that JNK was involved in regulating astrocyte activation under physiological conditions. Several studies demonstrate that p38MAPK also mediates the expression of GFAP and various astrocyte-regulated molecules in vivo and in vitro [64–67]. In this study, we applied the inhibitors of ERK, JNK, and p38 to inhibit the expression of p-ERK, p-JNK, or p-p38, which could, in turn, suppress the GFAP upregulation and improve the cell hypertrophy.

Moreover, Zhao et al. (2012) showed that Sirt1 downregulation induced by the pharmacological inhibitor salermide or RNA interference could attenuate the activation of ERK after brain injury. In addition, the inhibition of ERK activation could also decrease the Sirt1 expression, suggesting that there may be some interactions between Sirt1 and MAPK cascade activation.

In the present study, inhibition of ERK or JNK or p38 phosphorylation significantly upregulated the expression of Sirt1 and decreased the expression of GFAP in cultured cortical astrocytes and mice brain. The improved neurobehavioral function in ERK inhibitor, JNK inhibitor, or p38 inhibitor group indicated that these neuroprotective effects might be associated with alleviating the astrocyte activation. Interestingly, when the MAPK cascade activation was inhibited, the Sirt1 expression was significantly upregulated; however, when the overexpression of Sirt1 by pharmacological agonist (resveratrol) or Sirt1 interference was inhibited, the ERK activation was increased and the JNK and p38 activation were inhibited. These results further ravel some interactions between Sirt1 and MAPK activation after brain injury, although the precise mechanism remains to be further elucidated.

**Fig. 8:**
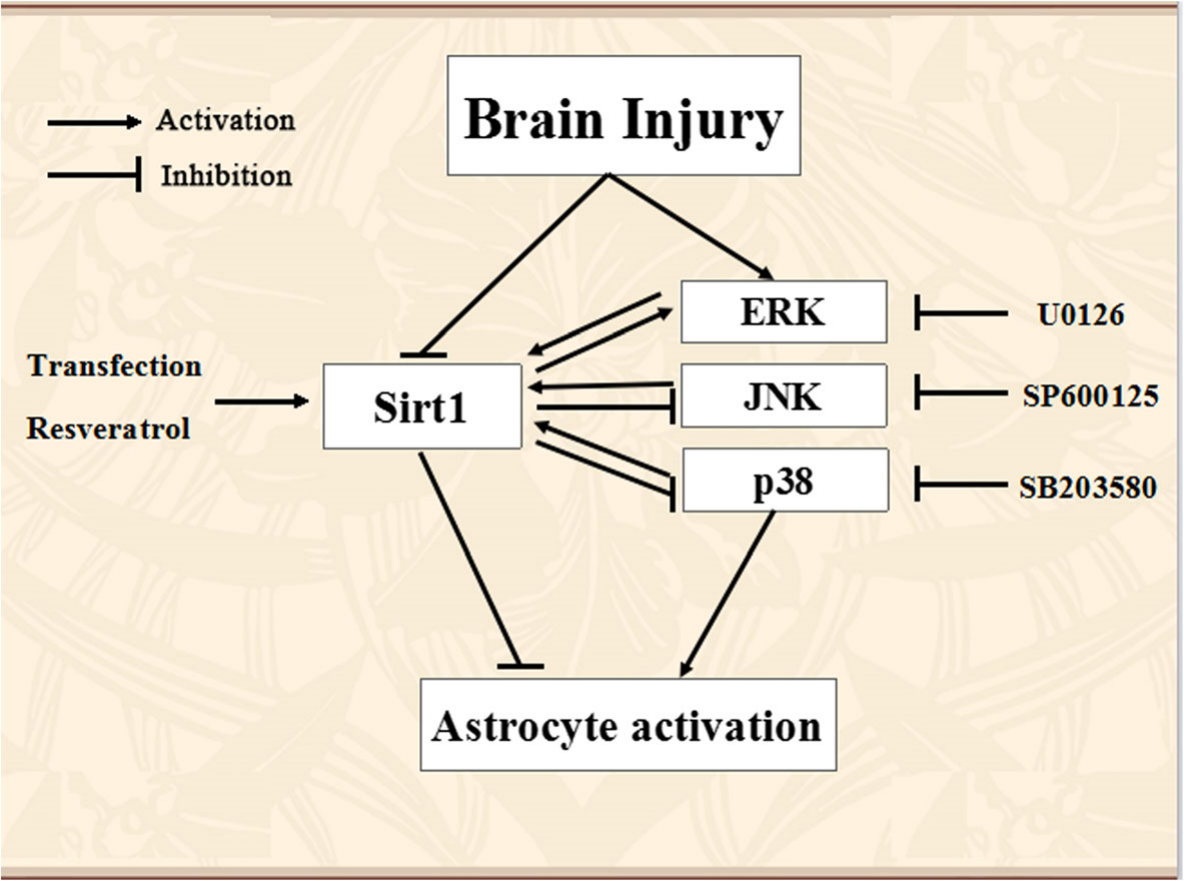
The possible interactions between Sirt1 and ERK, JNK, or p38MAPK pathway in traumatic brain injury. The brain injury reduced endogenous Sirt1 level and activated ERK, JNK, and p38MAPK pathways. Overexpressed Sirt1 attenuated astrocyte activation and decreased ERK, JNK, and p38MAPK activation. Inhibition of ERK, JNK, or p38MAPK activation increased Sirt1 expression and attenuated astrocyte activation

Herein, we have summarized the plausible dynamic relationship between Sirt1 and MAPK (ERK, JNK, and p38MAPK) pathways using a brain injury model in Fig. 8. The brain injury and IL-1β stimulation reduced Sirt1 and activated MAPK pathway leading to the astrocyte activation. The overexpression of Sirt1 by agonist or transfection-attenuated astrocyte activation regulated the MAPK pathway activation. The inhibition of MAPK pathway activation improved the astrocyte activation and elevated Sirt1 expression. Sirt1 interacted with MAPK activation via a synergic relationship to regulate astrocyte activation induced by brain injury in vivo or IL-1β stimulation in vitro, showing that both Sirt1 and MAPK pathways are potentially attractive therapeutic targets in the therapy of neurodegeneration induced by astrocyte activation. Thus, a new pharmacological product or endogenous method which could significantly elevate Sirt1 expression or activity might be a novel and efficient strategy for neuroprotection after brain injury.

## Conclusions

In summary, this study found that Sirt1 is an important topic for astrocyte activation induced by IL-1β stimulation in vitro and traumatic brain injury in vivo; inhibition of ERK, JNK, or p38MAPK activation suppressed GFAP upregulation and improved the cell hypertrophy to attenuate the astrocyte activation; Sirt1 interacted with ERK pathway activation via a synergic way, but an antergic way with JNK and p38MAPK pathway activation to mediate the expression of GFAP and astrocyte morphology that is the astrocyte activation. Investigation of the regulation and levels of these and other signaling molecules may be a novel and effective strategy for neuroprotection after brain injury.

## Abbreviations

CNS: Central nervous system
GFAP: Glial fibrillary acidic protein
IL-1β: Interleukin-1β
MAPK: Mitogen-activated protein kinase
p-ERK: Phosphorylated extracellular regulated protein kinases
p-JNK: Phosphorylated c-Jun N-terminal kinase
p-p38: Phosphorylated p38 activation
Sirt1: Silent mating type information
